# Functional comorbidities and brain tissue changes before and after lung transplant in adults

**DOI:** 10.3389/fncel.2022.1015568

**Published:** 2022-12-02

**Authors:** Matthew Scott Vandiver, Bhaswati Roy, Fahim Mahmud, Helen Lavretsky, Rajesh Kumar

**Affiliations:** ^1^Department of Anesthesiology and Perioperative Medicine, University of California, Los Angeles, Los Angeles, CA, United States; ^2^Department of Psychiatry and Biobehavioral Sciences, Semel Institute for Neuroscience and Human Behavior, University of California, Los Angeles, Los Angeles, CA, United States; ^3^Department of Radiological Sciences, University of California, Los Angeles, Los Angeles, CA, United States; ^4^Department of Bioengineering, University of California, Los Angeles, Los Angeles, CA, United States; ^5^David Geffen School of Medicine, Brain Research Institute, University of California, Los Angeles, Los Angeles, CA, United States

**Keywords:** perioperative neurocognitive dysfunction, depression, anxiety, delirium, magnetic resonance imaging

## Abstract

**Background:**

Adults undergoing lung transplant, as a lifesaving treatment for end stage lung disease, exhibit high levels of peri-operative neurocognitive dysfunction in multiple domains, including delirium, cognition, and autonomic deficits. These complications impact healthcare costs, quality of life, and patient outcomes. Post-operative symptoms likely result from loss of brain tissue integrity in sites mediating such regulatory functions. Our aim in this study was to examine peri-operative neurocognitive dysfunction and brain tissue changes after lung transplant in adults.

**Methods:**

We retrospectively examined the UCLA lung transplant database to identify 114 lung transplant patients with pre-operative clinical and neurocognitive data. Of 114 patients, 9 lung transplant patients had pre- and post-transplant brain magnetic resonance imaging. Clinical and neurocognitive data were summarized for all subjects, and brain tissue volume changes, using T1-weighted images, before and after transplant were examined. T1-weighted images were partitioned into gray matter (GM)-tissue type, normalized to a common space, smoothed, and the smoothed GM-volume maps were compared between pre- and post-transplant (paired t-tests; covariate, age; SPM12, *p* < 0.005).

**Results:**

Increased comorbidities, including the diabetes mellitus (DM), hypertension, kidney disease, and sleep disordered breathing, as well as higher rates of neurocognitive dysfunction were observed in the lung transplant patients, with 41% experiencing post-operative delirium, 49% diagnosed with a mood disorder, and 25% of patients diagnosed with cognitive deficits, despite incomplete documentation. Similarly, high levels of delirium, cognitive dysfunction, and mood disorder were noted in a subset of patients used for brain MRI evaluation. Significantly decreased GM volumes emerged in multiple brain regions, including the frontal and prefrontal, parietal, temporal, bilateral anterior cingulate and insula, putamen, and cerebellar cortices.

**Conclusion:**

Adults undergoing lung transplant often show significant pre-operative comorbidities, including diabetes mellitus, hypertension, and chronic kidney disease, as well as neurocognitive dysfunction. In addition, patients with lung transplant show significant brain tissue changes in regions that mediate cognition, autonomic, and mood functions. The findings indicate a brain structural basis for many enhanced post-operative symptoms and suggest a need for brain tissue protection in adults undergoing lung transplant to improve health outcomes.

## Introduction

Chronic lung diseases affect over 35 million people in the United States each year, with escalating healthcare costs of over $50 billion per year (Yelin et al., 2002; European Respiratory Society, 2017). For majority of patients, lung transplant remains the only viable treatment option for severe end-stage lung disease (Maxwell et al., 2015; Keller et al., 2019). Over 2,000 adults each year undergo lung transplant and contribute several billion dollars more in post-surgery healthcare costs (Chambers et al., 2018; Keller et al., 2019). In addition to increased survival, lung transplant can dramatically improve quality of life (Smeritschnig et al., 2005; Cohen et al., 2014). However, many challenges exist for patients after lung transplant, and despite significant advancement in clinical management strategies, the post-transplant survival continues to be lower (over 6 years), with few factors adding significant predictors of long-term risks (van der Mark et al., 2020).

Several factors, including the development of perioperative neurocognitive dysfunction (PNCD) and pre-operative comorbidities can affect post-transplant outcomes (Smith et al., 2018a). Patients with significant preoperative cognitive dysfunction and dementia are disqualified for lung transplant at most centers. PNCD encompasses cognitive impairment existing preoperatively, postoperative delirium, delayed neurocognitive disorder, and postoperative neurocognitive disorder that likely stem from multifaceted interactions between patients’ underlying comorbid susceptibilities and surgical factors (Moller et al., 1998; Vacas et al., 2013; Vandiver and Vacas, 2020). However, many centers do not routinely utilize a thorough battery of cognitive testing preoperatively to assess cognitive dysfunction, and thus, limited data are available. In addition, several comorbidities, including the diabetes mellitus (DM), hypertension, kidney disease, and sleep disordered breathing are common in adults undergoing lung transplant. Such comorbidities may enhance post-operative neurocognitive dysfunction that occur in over 50% of cases (O’keeffe and Ni Chonchubhair, 1994; Dyer et al., 1995; Abildstrom et al., 2000; Lemstra et al., 2008; Whitlock et al., 2011; Hood et al., 2018; Altay et al., 2020), but the exact underlying mechanisms remain poorly understood (Cupples and Stilley, 2005; Cohen et al., 2014; Smith et al., 2018a; Sommerwerck et al., 2021).

End stage lung disease leads to chronic hypoxic states that result in brain tissues changes, which are known to occur in other conditions with chronic hypoxia (Kumar et al., 2008, 2011, 2012, 2014; Macey et al., 2008; Pike et al., 2021; Roy et al., 2021a). Multiple brain areas are included in cognition control, including the prefrontal cortices, caudate, and hippocampus, which are involved in cognitive deficits post injury (Rubin et al., 2014; Pike et al., 2018; Roy et al., 2021a). Although the role of particular brain sites in PNCD is unclear, recent invasive and non-invasive imaging studies show compromised global gray matter volume within the postoperative period in patients that develop cognitive deficits, without determining precise regions of damage (Forsberg et al., 2017; Kant et al., 2017). While multiple reports enumerate neurologic complications after lung transplant, there is a paucity of imaging studies investigating brain tissue changes following lung transplant. Such investigation may shed light upon the mechanisms underlying cognitive dysfunction post-transplant.

To this end, MRI based diffusion tensor imaging (DTI) has been utilized to examine non-invasively regional brain neuro-inflammatory-induced brain tissue changes (Benveniste et al., 1992; Pierpaoli et al., 1993, 1996a; Matsumoto et al., 1995; Ahlhelm et al., 2002). Using DTI, several quantitative indices, including mean diffusivity (MD), can be calculated, which is commonly used to evaluate brain tissue injury (Basser and Pierpaoli, 1998; Le Bihan et al., 2001). MD measures average water diffusion within tissue (Basser et al., 1994; Pierpaoli et al., 1996b), and detects tissue pathology (Matsumoto et al., 1995; Warach et al., 1996; Ahlhelm et al., 2002; Chan et al., 2009). MD of water within tissue is influenced by the presence of tissue barriers (Le Bihan et al., 1991), and extra-cellular/extra-axonal fluid. In acute neuro-inflammation and tissue changes, neuronal and axonal swelling increase tissue barriers, reduce extracellular volume, and escalate cytotoxic edema; all factors contributing to reduced MD values (Matsumoto et al., 1995; Warach et al., 1996; Ahlhelm et al., 2002; Chan et al., 2009), as shown in animal models accompanying hypoxia (Pierpaoli et al., 1993; Tao et al., 2012). Since adults undergoing lung transplant will accompany neuro-inflammation, MD procedures may help identify PNCD and postoperative cognitive regulatory brain changes in adults with chronic lung disease.

Our study aim was to retrospectively assess the presence of PNCD and other comorbidities in an adult population that had undergone lung transplantation. We hypothesized that these patients would exhibit high levels of comorbidities and patients would reveal structural brain changes, based on magnetic resonance imaging, in cognitive regulatory areas explaining clinical findings.

## Materials and methods

Using the UCLA transplant database, we examined 114 adult patients admitted between Years 2009 and 2020 for inclusion in this study. We obtained clinical, demographic, and physiologic data from all patients. Of 114 patients, we identified 9 adult patients that had undergone lung transplant and had brain MRI scans both before and after lung transplant. The MRI data for 9 subjects were downloaded, as well as demographic, medical co-morbidities, Confusion Assessment method for the ICU (CAM-ICU), documented psychiatric or mood disorder, and any documentation of cognitive dysfunction following surgery were obtained. Demographic, physiologic, and primary lung pathology information are summarized in Table 1, and the presence of delirium, documented mood disorder, and any documentation of cognitive deficits after lung transplant are tabulated in Table 2.

**TABLE 1:** Demographics and other variables of the study population.

Variables	All patients number (%), [StDev]	MRI analysis group number (%), [StDev]
Age (years)	58.4 ± 11.4	65.1 ± 7.1
Sex (male, %)	66 (58%)	5 (55%)
BMI (kg/m^2^, mean ± SD)	24.2 ± 4.7	24.3 ± 4.0
**Pulmonary diagnosis (number, %)**		
IPF/ILD	67 (58.7%)	5 (56%)
COPD/Emphysema 11 (9.6%)	2 (22%)	
Hypersensitivity pneumonitis	6 (5.3%)	1 (11%)
Connective tissue diseases	13 (11.4%)	1 (11%)
Alpha 1 anti-trypsin disease	5 (4.4%)	
Bronchiolitis obliterans/PNA	4 (3.5%)	
PAH/Vascular	4 (3.5%)	
Cystic fibrosis	4 (3.5%)	
**Comorbidities**		
DM	42 (37%)	2 (22%)
HTN	70 (61%)	5 (56%)
CKD	51 (45%)	3 (33%)
Obesity	9 (8%)	1(11%)
OSA	17 (15%)	0 (0%)

StDev, standard deviation; BMI, body mass index; IPF, interstitial pulmonary fibrosis; COPD, chronic obstructive pulmonary disease; PAH, pulmonary arterial hypertension; DM, diabetes mellitus; HTN, hypertension; CKD, chronic kidney disease; OSA, obstructive sleep apnea.

**TABLE 2:** Documented mood and cognitive disorders in the study population.

Variables	Patients number (%), [StDev]	MRI analysis group number (%), [StDev]
Mood disorder	53 (49%)	7 (79%)
Delirium	[20/50] (41%)	[4/9] (44%)
Documented cognitive deficits	28 (25%)	4 (44%)

StDev, standard deviation.

### Magnetic resonance imaging

Brain imaging data were collected using a 1.5- or 3.0-Tesla MRI scanner (Siemens or GE). T1-weighted image series were acquired using the following parameters: repetition-time = 250–2,000 ms; echo-time = 2.43–15 ms; number of averages = 1/2; flip angle = 8–90°; matrix size = 192–313 × 256–384; field-of-view = 178–256 mm^2^ × 220–256 mm^2^; echo train length = 0/1; bandwidth = 81–320 Hz; slice thickness = 0.9–5 mm. We visually assessed T1-weighted images of all subjects for any major pathology, such as cystic lesions, infarcts, or tumors to subsequently exclude subjects, if found with any abnormality. For any head-motion related or other imaging artifacts, we critically examined T1-weighted images, and excluded the subject if any motion-related or other artifacts were found. All 9 adult patients’ MRI data before and after lung transplant were free from motion artifacts or any serious brain pathology.

### Data processing and analyses

The statistical parametric mapping package (SPM12),^1^ MRIcroN, and MATLAB-based (The MathWorks Inc., Natick, MA, USA) custom software were used for data processing and analyses. T1-weighted images were partitioned into gray matter, white matter, and cerebrospinal fluid tissue types. The Diffeomorphic Anatomical Registration Through Exponentiated Lie algebra algorithm (DARTEL) toolbox (Ashburner, 2007) was used to generate the flow field maps, which are non-linear deformations applied for warping all the gray matter images to match each other and template images that were implemented for normalization of gray matter maps to Montreal Neurological Institute (MNI) space (voxel size: 0.46 mm^3^ × 0.46 mm^3^ × 6 mm^3^). The modulated and normalized gray matter maps were smoothed using a Gaussian filter, and the smoothed gray matter (GM) maps were used for further statistical analyses.

### Background image

The average T1-weighted images from one control subject were normalized to MNI space. The normalized images were used as background images for structural identification.

### Region-of-interest analyses

Region-of-interest (ROI) analyses were performed to calculate regional gray matter volumes to determine magnitude differences between groups. The ROI values were extracted using the regional masks of specific brain regions that appeared in whole brain analyses and smoothed gray matter maps of all subjects.

### Statistical analyses

#### Demographics, clinical, and neuropsychologic variables

The Statistical Package for the Social Sciences (IBM SPSS, v28.0, Armonk, NY, USA) was used for assessment of demographic, physiologic, mood, cognition, and clinical variables. A *p* < 0.05 was considered significant.

#### Regional brain gray matter volume changes after lung transplant

For assessment of regional brain GM volume changes, the smoothed whole-brain gray matter maps were compared between pre- and post- lung transplant using paired t-tests (SPM12; covariate, age; uncorrected *p* < 0.005). Brain clusters with significant GM volume differences between pre- and post-transplant were superimposed onto the background images for structural identification.

#### Regional brain gray matter volumes

Regional gray matter volumes, calculated from ROI analyses, were examined for significant differences between pre- and post- lung transplant using repeated measure ANOVA (SPSS; covariates, age). A *p*-value of <0.05 was chosen to establish statistical significance.

## Results

### Demographics, clinical, and neuropsychologic variables

Demographics and clinical data of lung transplant patients were summarized in Table 1. Mean age of adults undergoing transplant was 58 years, and 58% subjects were males. Also, mean body-mass-index (BMI) was 24.2 ± 4.7 kg/m^2^ with lower (8%) obesity rate. Of 114 adults, 42 patients (37%) had type 2 diabetes mellitus, 70 (61%) hypertension, 51 (45%) chronic kidney disease, and 17 (15%) were diagnosed with obstructive sleep apnea (OSA). In addition, majority (58.7%) of lung transplant patients have interstitial pulmonary fibrosis, with 9.6% having a primary diagnosis of chronic obstructive pulmonary disease (COPD)/Emphysema, 5.3% hypersensitivity pneumonitis, 11.4% connective tissue disease, 4.4% Alpha-1-Anti-Trypsin disease, 3.5% Bronchiolitis Obliterans, 3.5% pulmonary arterial hypertension, and 3.5% cystic fibrosis.

Perioperative neurocognitive dysfunction and mood disorders are summarized in Table 2. Of 114 patients, delirium emerged in 50 (41%) patients, cognitive deficits were diagnosed or were worse in 28 (25%) following transplantation, and mood disorders noted in 53 (49%) adults. Severity of mood disorder was not recorded for these patients in the medical record. Delirium was assessed utilizing CAM-ICU scores, and to assess for cognitive dysfunction, we investigated the electronic medical record (EMR) for any diagnoses from the patient’s primary care provider, advanced lung disease physician (pulmonologist), psychiatrist, or formal cognitive testing which included documentation of cognitive deficits. At least 21 (18%) of the subjects had documented autonomic dysfunction, such as gastroparesis, excessive sweating, bulbar dysfunction, orthostasis, or unexplained tachycardia, although much of the data for these diagnoses are lacking from patients prior to 2015. Mood disorders were investigated mainly with diagnoses of anxiety disorder and or depression by the patient’s primary care provider, advanced lung disease physician (pulmonologist), psychiatrist, or other consultant physician.

Demographic data from adults with pre- and post- lung transplant and MRI are summarized in Table 1. Mean age of adults is 65.1 years, with 56% subjects as male, BMI 24.3 ± 4.0 kg/m^2^ with an average of 3.9% reduction in BMI from pre- to post-op MRI. Of 9 adults, 2 (22%) patients have type 2 diabetes mellitus, 5 (56%) hypertension, 3 (33%) chronic kidney disease, and 1 (11%) diagnosed with OSA. EMR analysis revealed delirium in 4 (44%) patients with documented cognitive deficits post-operatively in 4 (44%), and autonomic dysfunction and mood disorder in 7 (78%) patients (Table 2).

### Regional brain gray matter volume changes after lung transplant

**FIGURE 1:**
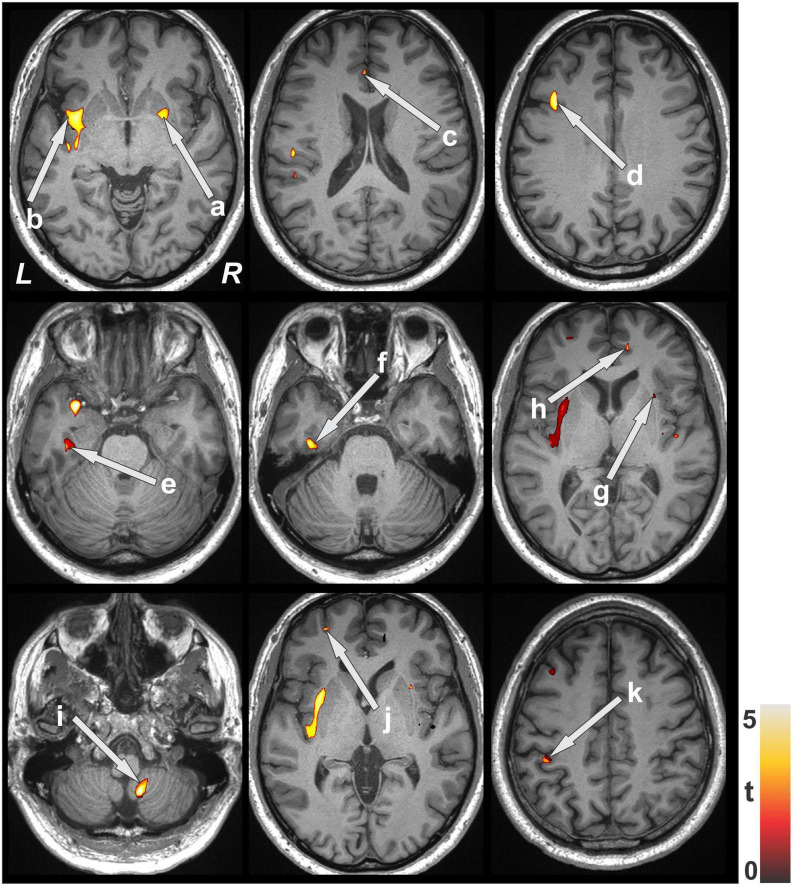
Brain regions with decreased gray matter volume after lung transplant over pre- lung transplant. Brain clusters with significant GM volume differences between pre- and post-transplant were superimposed onto the background image for structural identification. Sites pointed with arrows showing reduced gray matter volume included the putamen (a), insular cortices (b,g), anterior cingulate (c,h), frontal cortices (d), para-hippocampal gyrus (e), temporal cortices (f), cerebellum (i), prefrontal cortices (j) and parietal cortices (k). All images are in neurological convention (L, left; R, right). Color bar indicates t-statistic values.

**FIGURE 2:**
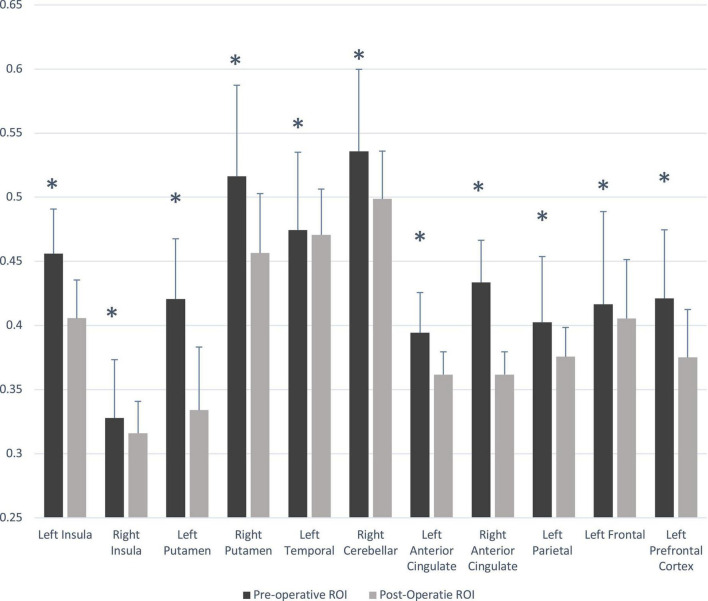
Gray matter volume changes in pre- and post- lung transplant patients. Significant difference between group (*p* < 0.05) is marked with asterisk (*). ROI, region of interest.

Multiple brain areas showed reduced relative regional gray matter volume after lung transplant compared to pre- lung transplant (Figure 1), and those sites included the bilateral putamen (a), bilateral insular cortices, bilateral anterior cingulate (c, h), left frontal cortex (d), para-hippocampal gyrus (e), left temporal cortex (f), right cerebellum (i), left prefrontal cortex (j), and left parietal cortex (k). Only right temporal cortex showed a slightly increased gray matter volume in post lung-transplant compared to pre- lung transplant adults. ROI analysis was performed and is shown in Figure 2 and Table 3. This includes GMV changes in mm^3^ with associated *p*-values (all <0.02). These regions of interest show decreases in GMV between pre- and post-operative MRI with varying effect sizes based on region. Given recent publications showing that BMI can affect GMV in individuals (Fernández-Andújar et al., 2021; Hidese et al., 2022; Li et al., 2022b), we performed a supplemental analysis (Supplementary Figure 1), integrating BMI changes, age, and sex as covariates. Regions identified with GMV changes were mostly unaffected, with slight decreases or increases of clusters.

**TABLE 3:** Regional brain gray matter volume values (*n* = 8, mean ± SD, mm^3^) from pre- and post-lung transplant patients.

Brain regions	Pre-operative	Post-operative	*P*-values
Left insula	0.456 ± 0.098	0.406 ± 0.085	0.001
Right insula	0.328 ± 0.129	0.316 ± 0.070	0.015
Left putamen	0.421 ± 0.133	0.334 ± 0.139	0.011
Right putamen	0.516 ± 0.201	0.457 ± 0.131	0.013
Left temporal	0.474 ± 0.171	0.471 ± 0.101	0.005
Right cerebellar	0.536 ± 0.181	0.499 ± 0.105	0.001
Left anterior cingulate	0.394 ± 0.089	0.362 ± 0.050	0.007
Right anterior cingulate	0.434 ± 0.093	0.362 ± 0.050	0.003
Left parietal	0.403 ± 0.145	0.376 ± 0.064	0.005
Left frontal	0.416 ± 0.205	0.406 ± 0.130	0.006
Left prefrontal cortex	0.421 ± 0.151	0.375 ± 0.106	0.001

SD, standard deviation.

## Discussion

In this study, preoperative lung transplant patients show multiple comorbidities, including type 2 diabetes mellitus, hypertension, chronic kidney disease, and OSA, with the primary lung pathology interstitial pulmonary fibrosis, COPD/Emphysema, hypersensitivity pneumonitis, connective tissue disease, Alpha-1-Anti-Trypsin Disease, Bronchiolitis Obliterans, pulmonary arterial hypertension, and cystic fibrosis. In addition, significant perioperative neurocognitive, mood, and autonomic dysfunction, including delirium, cognitive deficits following transplantation, and anxiety/depression were documented. Adults after lung transplant showed reduced regional gray matter volume in multiple brain areas, including the putamen, insula, anterior cingulate, frontal, parietal, and temporal cortices, para-hippocampal gyrus, and cerebellum, regions that mediate mood, autonomic, and cognitive functions, which may have contributed to postoperative functional deficits.

It should be noted that although voxel based analysis of gray matter volume changes have been used extensively to study a variety of neurologic disorders (Lavretsky et al., 2008; Popescu et al., 2015; Yankowitz et al., 2021; Li et al., 2022a; Long et al., 2022), the underlying mechanisms for these gray matter volume changes is unclear. The GMV changes have been correlated in multiple studies with cellular metrics in mice utilizing MRI, two-photon *in vivo* microscopy, miRNA and immunohistochemical analysis in neurodegenerative and mood disorder models of mice showing physical tissue volume, cellular number, nearest neighbor distance, nucleus volume, and a host of smaller contributors, such as spine density and plasticity (Khan et al., 2020; Asan et al., 2021). Additionally multiple studies have shown morphologic cellular changes in post mortem studies in mood and cognitive disorders (Rajkowska, 2000; Stockmeier et al., 2004; Van Otterloo et al., 2009; Rubinow et al., 2016; Rajkowska et al., 2017; Anderson et al., 2020). These studies show different patterns of cell loss, cellular atrophy (Rajkowska, 2000), length of axons (Rajkowska et al., 2017), and increased cell number in differing conditions and locations. Thus, neuronal loss secondary to hypoxia is only one hypothesis for GMV changes in this population. However, all of these possible contributors may affect cognition, and this demonstrates the need for future studies with biochemical components that may better tease out the underlying neurologic architecture changes, leading to worsening cognition. We still note, however, that GMV has been extensively associated with changes in many and disorders as described above and even in specific traits such as respect (Nakatani et al., 2020). Aging related changes in brain volume may confound studies utilizing MRI imaging (Asan et al., 2021), however, keeping the scan times close together may alleviate this potential confounder allowing for the contribution from surgery to be assessed more clearly. Additionally, other co-morbidities and even BMI (Fernández-Andújar et al., 2021; Hidese et al., 2022; Li et al., 2022b) will affect gray matter volume as we have demonstrated, which necessitates close control of patient selection and attention to changes in their overall health status before and after their lung transplant.

Post lung transplant adults are at a significantly elevated risk of cognitive dysfunction and a myriad of poor neurologic outcomes after their transplant. Previous observational studies have demonstrated deficits in cognition, including learning, memory, and executive function, all of which are vital to perform activities of daily living and the maintenance of wellness (Hoffman et al., 2012; Smith et al., 2018a,b). Several other diseases with respiratory compromise, such as cystic fibrosis, chronic obstructive pulmonary disease, obstructive sleep apnea, and asthma demonstrate altered cognition and mood (Crews et al., 2001; Dodd et al., 2010; Dodd, 2015; Roy et al., 2021b), and suggest that these disorders might be seen with even high frequency in the end-stage lung disease adults requiring lung transplant. Our study findings demonstrate a sizeable amount of PNCD in this lung transplant population, which are likely underdiagnosed, given the lack of standardized formal testing in the postoperative period. Our adults also exhibited large amounts of mood and anxiety symptoms (∼50%), as well as autonomic dysfunction. We also demonstrated that while OSA is generally similar in prevalence to the general adults (Senaratna et al., 2017), we note relatively high percentages of hypertension and chronic kidney disease, likely related to the events leading up to surgery and beyond. Although these conditions are relatively mild in the preoperative period, since severe comorbidities are disqualifying for acceptable transplantation status, the surgery induced stress, immunocompromising steroids and medications, and patients’ operative course likely contribute to new comorbidities or worsening conditions after surgery. In addition, these comorbidities also likely contribute to worse post-operative outcomes and may contribute to higher rates of PNCD, larger sample size and prospective studies would be required to identify each comorbidity contribution to overall increase in PNCD. Also, we report very low levels of obesity compared to the general population; however, this is most likely related to standard cut-offs for BMI in lung transplant and the disease process that often limits patients ability to gain weight with morbid obesity being a disqualifying factor for most transplant centers. The data suggest that this is a critical problem faced by post-lung transplant adults, as they navigate life after surgery and the desperate need to assess and address any cognitive deficits prior to their escalation to a problem easily identified by impact on daily life activities.

Here we report significantly reduced gray matter volumes in multiple brain areas after lung transplant adults, including the cerebellum, putamen, temporal, frontal, and prefrontal cortices, areas that are highly involved in cognition, mood, and autonomic functions. Injury in the prefrontal cortex has adverse implications in planning complex cognitive behavior, personality expression, decision making, and moderating social behavior (Frith and Dolan, 1996). The hippocampus, a crucial site involved in cognition, has extensive connections with the prefrontal cortex, including direct reciprocal connections between the medial prefrontal cortex and the medial temporal lobe (Rubin et al., 2014) and changes in this area have been associated with pathological states such as major depressive disorder (Stockmeier et al., 2004).

While neuronal damage and morphological changes are suggested as possible primary mechanisms in end-stage lung diseases, hypoxia and hypercapnia are also present in this patient population and likely contribute to poor outcomes through a variety of mechanisms as discussed above. While one might posit that many impaired functions, especially mood disturbances and cognitive deficits in this patient population may result from many outside aspects of a patient’s life in end-stage lung disease, our findings demonstrate a brain structural origin for their symptomatology. Multiple prior studies demonstrated that the prefrontal cortex, hippocampus, and amygdala comprise a network for mood regulation (Soares and Mann, 1997; Drevets, 2000), and in our study, part of these regions showed decreased regional gray matter volume. Anxiety and fear response are known to be affected by dopamine levels in the nucleus accumbens, a region which is regulated by the amygdala. It has been established that the insula and cingulate regions are involved in a network that regulates behavioral responses, including mood regulation, and both insula and cingulate areas are injured here.

Another region that we identified with reduced gray matter volume in adults after lung transplant was the cerebellum. Cerebellar climbing fibers are crucial for functional possesses, and tissue changes can lead to disruption in motor performance, as well as autonomic dysfunction (Miura and Reis, 1969; Hirano, 2013). In our patient population, over 40% adults showed some form of autonomic abnormality after lung transplant, which is not surprising given the impressive tissue changes post-transplant in those regulatory areas. In addition to motor and autonomic functions, the cerebellum also contributes to cognitive function via visuospatial processing and executive functioning. There are both feed-forward and feed-backward connectivity in this respect and further provides a foundation for the understanding of cognitive deficits in this patient population.

Altered gray matter regions that we discovered in our study of lung transplant adults likely play an important role in their cognition, mood, and general wellbeing. High levels of anxiety, depression, and impaired cognition can greatly affect patient management given that adherence to immunosuppression, follow-up at outpatient clinics, the ability to exercise, and general daily activities; all affect outcomes from life-saving lung transplant procedures. Disruptions in these key areas in adults will lead to increased hospitalizations, increases in healthcare costs, and significant impacts on the quality of life (Riekert et al., 2007; Smith et al., 2010; Yohannes et al., 2012; Snell et al., 2014). These findings even more strongly suggest the need for cognitive screening, and especially the prompt recognition and treatment of depression and anxiety in these adults. More thorough identification of cognitive and mood deficits will lead to improvements in outcomes in an already difficult adult patient population.

Several study limitations warrant further investigation with bigger MRI sample size before and after lung transplant data. One of the biggest limitations of our study is the relatively small sample size with MRI data, making it difficult to correct for multiple comparisons, which may affect the findings. However, an excellent mitigating factor is that the use of the same adults for pre- and post-transplant comparisons with extended clusters threshold that shows subtle and accurate tissue changes. In addition, MRI data were collected with different scanners and image resolutions, possibly also leading to type 1 error. However, all brain MRI images were bias corrected and down-sampled to one resolution before voxel-based comparisons that reduces variability. Additionally, white matter damage and small ischemic strokes can also be responsible for symptoms of depression, apathy, anxiety and executive dysfunction as a large body of literature has shown (Lavretsky et al., 2008; Mueller et al., 2010), and while many of these changes are likely reflected in structural brain changes, larger sample size and higher resolution would help in differentiating specific causes for loss of gray matter volume. We mitigated the contribution for ischemic infarcts by excluding patients with diagnosed stroke on MRI. Another significant limitation of this study includes the lack of CAM-ICU data for many participants, which were transplanted before routine documentation of CAM-ICU scores in the electronic medical record. The retrospective nature of this study also limits our ability to control for scan timing which may introduce age related changes as a confounder, although we address this within the statistical analysis it does not address the underlying mechanism for gray matter volume loss as no biochemical or pathologic sample data is available for these patients and future studies may help address this point by the addition of biomarkers of neuronal cell death or inflammation tied to brain pathology. In addition, the patient population’s lack of both universal screening and nuanced assessment of cognition in these adults likely underestimates the number of patients with cognitive deficits as we were unable to utilize Montreal cognitive assessment or other standard screening tool, given the nature of retrospective analysis. Thus more longitudinal assessments of cognition and mood evaluation would further enhance the validity of tying specific brain regions that mediate impaired functions.

## Conclusion

Adults waiting for lung transplant show significantly increased comorbidities, including the diabetes mellitus, hypertension, kidney disease, OSA, and neurocognitive dysfunction before transplant. In addition, high levels of delirium, impaired cognition, and mood changes were observed in a subset of patients available for brain MRI evaluation. Adults after lung transplant showed significant brain structural changes, as evidenced by reduced gray matter volume, and these neuronal changes were localized in specific brain regions, including the frontal, prefrontal, parietal, and temporal cortices, anterior cingulate, insular, putamen, and cerebellar areas that mediate in cognition, autonomic, and mood regulation. The findings suggest a brain structural basis for neurocognitive dysfunction after lung transplant and demonstrate the need for early identification and treatment of deficits in these adults to improve outcomes.

## Data availability statement

The data analyzed in this study is subject to the following licenses/restrictions: The datasets analyzed during the current study are not publicly available since it contains patient’s personal health information but are available from the corresponding author on reasonable request. Requests to access these datasets should be directed to RK, rkumar@mednet.ucla.edu.

## Ethics statement

The studies involving human participants were reviewed and approved by Institutional Review Board of University of California, Los Angeles. Written informed consent for participation was not required for this study in accordance with the national legislation and the institutional requirements.

## Author contributions

MV: conception, data acquisition, analysis, interpretation of data, and drafted the work. BR: analysis, interpretation of data, and substantively revised the initial draft. FM: data acquisition and analysis. HL: interpretation of data and substantively revised it. RK: conception, data acquisition, interpretation of data, and substantively revised the initial draft. All authors read and approved the final manuscript.

## Footnotes

1
http://www.fil.ion.ucl.ac.uk/spm/

## Abbreviations

BMI: body-mass-index
DM: diabetes mellitus
CAM-ICU: confusion assessment method for the ICU
COPD: chronic obstructive pulmonary disease
EMR: electronic medical record
MNI: Montreal Neurological Institute
PNCD: perioperative neurocognitive dysfunction
SPM12: statistical parametric mapping package 12
SPSS: statistical package for the social sciences
OSA: obstructive sleep apnea.
