# Supplementary material for: Functional comorbidities and brain tissue changes before and after lung transplant in adults
Source: Front Cell Neurosci. 2022 Dec 2;16:1015568. doi: 10.3389/fncel.2022.1015568 (PMC9755201; doi:10.3389/fncel.2022.1015568)
Supplement: Supplementary file 1 [file Data_Sheet_1.PDF]

Supplemental Data:

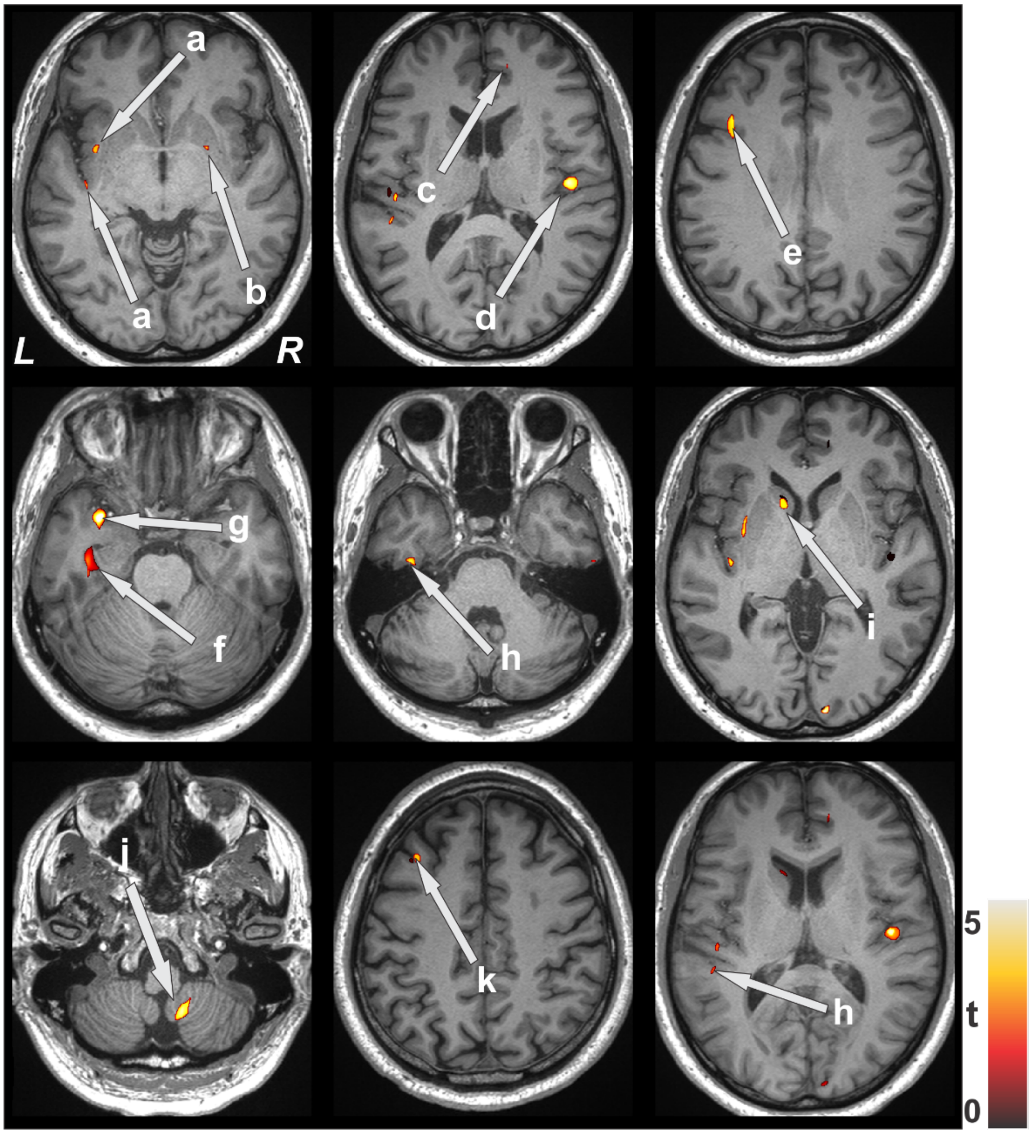

Figure Legend:

Figure S1: Brain regions with decreased gray matter volume after lung transplant over pre- lung transplant, accounting for age, sex, and BMI. Brain clusters with significant GM volume differences between pre- and post-transplant were superimposed onto the background image for structural identification. Sites pointed with arrows showing reduced gray matter volume included the insular cortices (a, d), putamen (b), anterior cingulate (c), frontal cortices (e, k), para-hippocampal gyrus (f), amygdala (g), temporal cortices (h), caudate (i), and cerebellum (j). All images are in neurological convention (L, left; R, right). Color bar indicates t-statistic values.
